# Epidermal grafting versus split-thickness skin grafting for wound healing (EPIGRAAFT): study protocol for a randomised controlled trial

**DOI:** 10.1186/s13063-016-1352-y

**Published:** 2016-05-17

**Authors:** Muholan Kanapathy, Nadine Hachach-Haram, Nicola Bystrzonowski, Keith Harding, Afshin Mosahebi, Toby Richards

**Affiliations:** Division of Surgery & Interventional Science, University College London, London, UK; Royal Free Hospital Wound Healing Group, Department of Plastic and Reconstructive Surgery, The Royal Free Hospital, London, UK; Cardiff University, Wound Healing Research Unit, School of Medicine, Heath Park, Cardiff, UK

**Keywords:** Epidermal graft, Split-thickness skin graft, CelluTome, Wound healing

## Abstract

**Background:**

Split-thickness skin grafting (SSG) is an important modality for wound closure. However, the donor site becomes a second, often painful wound, which may take more time to heal than the graft site itself and holds the risk of infection and scarring. Epidermal grafting (EG) is an alternative method of autologous skin grafting that harvests only the epidermal layer of the skin by applying continuous negative pressure on the normal skin to raise blisters. This procedure has minimal donor site morbidity and is relatively pain-free, allowing autologous skin grafting in an outpatient setting. We plan to compare EG to SSG and to further investigate the cellular mechanism by which each technique achieves wound healing.

**Methods/design:**

EPIGRAAFT is a multicentre, randomised, controlled trial that compares the efficacy and wound-healing mechanism of EG with SSG for wound healing. The primary outcome measures are the proportion of wounds healed in 6 weeks and the donor site healing time. The secondary outcome measures include the mean time for complete wound healing, pain score, patient satisfaction, health care utilisation, cost analysis, and incidence of adverse events.

**Discussion:**

This study is expected to define the efficacy of EG and promote further understanding of the mechanism of wound healing by EG compared to SSG. The results of this study can be used to inform the current best practise for wound care.

**Trial registration:**

Clinicaltrials.gov identifier, NCT02535481. Registered on 11 August 2015.

**Electronic supplementary material:**

The online version of this article (doi:10.1186/s13063-016-1352-y) contains supplementary material, which is available to authorized users.

## Background

Split-thickness skin grafting (SSG) is a current standard of care for wound closure for non-healing wounds. SSG involves excision of the epidermis and part of the dermis, leaving behind the reticular dermis in the donor site, which enables the skin to heal by secondary intention [1]. Despite SSG being an important modality for wound closure, the donor site becomes a second, often painful wound, which may take more time to heal than the graft site itself and holds the risk of infection and scarring.

Epidermal grafting (EG) is an emerging and promising option to overcome these challenges. Epidermal grafting is a method of autologous skin grafting that harvests only the epidermal layer of the skin from the donor site by applying gentle heat and continuous negative pressure on the normal skin to raise blisters [2, 3]. The roof of the blister, which is the epidermis, is then excised and transferred onto the wound. As the dermis in the donor site remains untouched, the skin regenerates itself without scarring. This procedure also causes minimum pain as the pain fibres in the dermis are unstimulated, allowing autologous skin grafting in the outpatient setting without the need for local anaesthesia.

This study evaluates the efficacy of EG using the CelluTome Epidermal Harvesting System (Acelity, San Antonio, TX, USA), an automated epidermal harvesting system that produces an array of epidermal micro-grafts. In a pilot study carried out in our centre using this system, EG was noted to be an effective method of autologous skin grafting with complete wound healing achieved in two-thirds of selected patients with minimal or no pain and a scar-free donor site [3]. The ability to perform EG in outpatient settings eliminates the need for a theatre space and a hospital bed, with potentially better patient satisfaction. However, it is not known if EG is an effective clinical alternative to SSG.

The mechanism of wound healing by EG may be different compared to SSG. EG is postulated to promote wound healing by expressing growth factors that accelerate wound healing and encourage keratinocytes to migrate from the wound edge [2]. We hypothesise that EG has similar wound healing rates to SSG at 6 weeks but with less donor site morbidity. We wish to evaluate the efficacy of EG as an alternative to SSG and to further investigate the mechanism by which each technique achieves wound healing.

## Methods/design

### Trial design

This study is a multicentre, randomised, control trial with two parallel groups. Eligible patients will be randomised to EG or SSG using a computerized randomisation method. This protocol is reported in accordance to the SPIRIT 2013 guideline (see Additional file 1) [4].

### Research ethics approval

This trial has approval from the National Research Ethics Service Committee London-Fulham (project ID: 15/LO/0556) and from the National Health Service Research & Development Department, Royal Free Hospital (project ID:9417). This trial is conducted in accordance to the Declaration of Helsinki and the recommendations of Good Clinical Practice.

### Study setting

Participants will be recruited at the Royal Free Hospital (RFH), London, and the University Hospital Wales, Cardiff. The Royal Free Hospital is an academic teaching hospital and is associated with the University College London (UCL).

### Eligibility criteria

Patients referred by consultant plastic surgeons for skin grafting are eligible for the study. Before enrolment, patients will be screened for inclusion in the trial, and a patient information sheet will be given. This process will include an explanation of the aims, methods of skin grafting and subsequent wound management, anticipated benefits, and potential hazards of the study. Patients are given sufficient time (offered a period of 24 hours or more if needed) to consider whether they wish to participate. Patients will then be offered participation in the study, and informed consent will be obtained (see Additional file 2). Treatment will be given within 7 days of patient enrolment.

Inclusion criteria are as follows:

1. Age ≥ 18 years
2. Wound measuring more than 1 cm x 1 cm and less than 6 cm x 6 cm (1 % total body surface area)
3. Clean, healthy granulating bed
4. Patients will be required to understand and be willing to participate in the trial and be able to comply with the weekly visits and follow-up regime

Exclusion criteria are as follows:

1. Infected wound
2. Wound at the plantar of the foot
3. Unsuitable for split-thickness skin grafting
4. Previous history of excessive bleeding associated with surgical biopsies or trauma
5. Uncontrolled diabetes mellitus, as measured by HbA1c ≥ 10 percent
6. Presence of one or more medical conditions including renal, hepatic, hematologic, active auto-immune or immune diseases
7. Use of systemic steroid or immunosuppressant
8. Not fit for surgery (ASA classification ≥ 4)

### Interventions

#### Wound bed preparation

All wounds will be prepared per normal clinical practise, which is either using the negative pressure wound therapy (NPWT) or appropriate wound dressings to achieve a healthy granulating bed. Wound swabs will be performed to ensure no bacterial growth. During the time of wound bed preparation, the patient will be referred to the research team. When the wound bed is deemed ready for grafting, as agreed on by two treating clinicians, patients will then be screened and offered a patient information sheet for inclusion in the trial. Once the patients are ready for intervention, following review by the study team, patients will undergo informed consent and randomisation.

#### Epidermal graft

Prior to grafting, the wound will be cleaned using wound irrigation solution by the surgeon and debrided if necessary. The suction head of the CelluTome Epidermal Harvesting System will be applied to the donor site (thigh) for 30–40 minutes to harvest the epidermal graft per protocol [3, 5]. The harvested epidermal grafts will then be transferred onto the wound using a non-adhering silicone dressing (Adaptic Touch, Systagenix, Airebank Mills, Skipton, UK). The wound is then dressed with gauze or NPWT, as deemed appropriate by the treating clinician based on the type of the wound. The use of iNadine (Systagenix) will also be considered over the Adaptic Touch for wounds that are more exudative or had previous infections. The dressing will be secured with a crepe bandage or a Mepore dressing (Mölnlycke Health, Dunstable, Bedfordshire, UK). The donor site will be dressed with Tegaderm (3M). The wound and donor site will be reviewed on day 7 ± 2 post-grafting.

#### Split-thickness skin graft

Patients will undergo this procedure in the operating theatre under general or local anaesthesia. The wound will be initially debrided by the treating surgeon in a similar manner to the EG group. Skin will be harvested from the thigh using an electric air dermatome, set to cut at the thickness of 8–10/1000 inch, which will then be meshed by 1:1.5. The wound will be grafted and dressed with Adaptic Touch (Systagenix), gauze, and a Mepore or wool and crepe bandage, depending on the site of the graft. The donor site will be dressed with Kaltostat (Alginate dressing) with a 2.5-cm overlay beyond the wound margins and secured with Mefix. Per standard clinical practise, the graft will be checked at day 7 ± 2.

#### Wound exudate sampling and punch biopsy

Wound exudate sampling will be performed by applying a filter paper (Whatman™ qualitative filter paper) on the wound for 15 minutes until it is moist. The filter paper will then be stored in a sterile vial and transferred to the laboratory. The wound fluid sampling will be performed before grafting and at each weekly review.

Skin punch biopsies (4 mm) will be taken from two locations, at the centre of the wound and at the wound edge, after administering adequate local anaesthesia (2 % lidocaine). This procedure will be done prior to grafting and repeated at day 7 post-grafting. The specimens are then placed in a sterile vial containing 4 % paraformaldehyde and transferred to the laboratory.

#### Laboratory studies methodology summary

The methodology is summarized as follows:

1. The wound exudate samples will be used to determine the type and concentration of growth factors, pre-grafting and post-grafting, using an enzyme-linked immunosorbent assay (ELISA) [6].
2. The skin biopsies will be used to compare the expression pattern of keratinocyte proliferative markers and Connexin protein (gap junctional proteins) before and after grafting at the wound edge and the centre of the wound. Tissues will be cryosectioned and stained with haematoxylin and eosin (H&E) and analysed for immunohistochemistry [7].

### Study outcome

The co-primary endpoints are the proportion of wounds with complete healing at 6 weeks post-grafting and the time for donor-site healing. Complete wound healing is defined as 100 % re-epithelialisation. The assessment of wound healing will be done via wound measurement at each review. The three-dimensional photographs of the wounds and the donor sites will be taken at each weekly visit using a LifeViz 3D camera (from Quanticare or similar) to obtain high-quality, accurate, and standardised images for digital measurement of the wound surface area [8]. These images will be stored in the patient’s digital photo diary. An independent blinded analysis of the photo diary will be carried out by two plastic surgeons. Inter-rater and intra-rater reliability will also be assessed. The wounds and the donor sites will be assessed using the PUSH tool, a standardised wound assessment tool, at each visit [9]. The PUSH scores for the wounds and the donor sites will be statistically analysed.

The secondary endpoints include the mean time for complete wound healing; pain score as reported by the patients using a numerical rating scale (scale of 0–10); patient satisfaction measured using a validated patient skin graft satisfaction questionnaire [10]; healthcare utilisation; and cost analysis measured by the consumables used, the frequency of visits, and the incidence of adverse events. The incidence of serious adverse events (SAEs) include mortality of any cause within the 3-month duration from the time of initial therapy, the incidence of device-related adverse events (DAEs), and the incidence of wound-related adverse events (WAEs) occurring within the study duration. The patient skin graft satisfaction questionnaire will be completed by the participants at the 6-week and 3-month visit.

Furthermore, we will determine the wound-healing mechanism of EG compared to SSG by analysing the type and concentration of growth factors expressed by the grafts, as well as the expression of Connexin proteins (gap junctional protein) and keratinocyte proliferative markers at the wound edge and the centre of the wound before and after grafting.

### Participant timeline

**Fig. 1:**
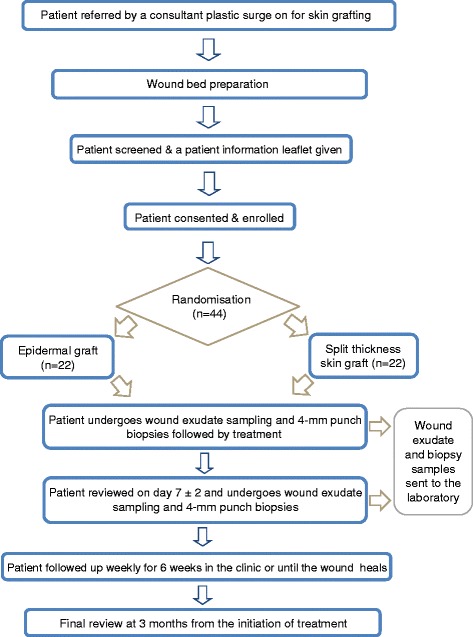
Flow chart illustrating patient’s journey throughout the study

The study was opened to recruitment in October 2015 and is anticipated to close in September 2017. Each patient will be followed up weekly for 6 weeks or until the wound heals. The final review will be at the third month from the initiation of the treatment. If the primary intervention had failed at week 6 ± 2, re-grafting and repeat of biopsy as per protocol will be considered after discussing with patient. Failed intervention is defined as increasing wound size or failure of 50 % reduction in wound size at week 6 ± 2. Figure 1 summarises the patient’s journey throughout the trial.

### Sample size

Our pilot study revealed that both techniques offer the same healing rate at 6-weeks post-grafting; however, the donor site morbidity is present in 40 % of the patients with split-thickness skin graft while only 5 % is seen in patients with the epidermal graft. Donor site morbidity includes discolouration, scarring, pain, and risk of infection.

Given a significance level of 0.05 for 80 % power, a sample size of 19 patients per group is yielded. In consideration of a potential dropout rate of 15 %, adjustments have been made to the sample size, with an increase to 22 patients per treatment arm. A total of 44 patients will be recruited into the study. The timeline for recruitment is 24 months.

### Randomisation, allocation concealment, and blinding

Once consented, patients will be randomly assigned to one of the treatment groups. A random allocation sequence will be computer generated using SPSS version 22 (IBM, Armonk, NY, USA). The allocation sequence will be sealed in identical opaque envelopes and given to the enrolling researcher upon receipt of patient consent. The surgical team, clinical staff, and patient will not be blinded to the intervention status.

### Data collection and management

All data collected will be recorded on paper forms and in a digital clinical research folder (CRF). Data will be collected by the surgical team and trial personnel. A research fellow will ensure the accuracy of the data collection by performing sample assessments at regular intervals. Any adverse events will be recorded and reported to the primary investigators and the institutional ethics committee.

Wounds will be assessed and recorded in a wound assessment form at each visit. Details on patient’s co-morbidities, wound duration and type, and previous wound bed-preparation methods will be recorded. The three-dimensional photographs will be used to measure the wound surface area digitally. The number and cost of outpatient visits will be recorded, and the type and cost of the dressings used will be documented.

### Data storage

The multidisciplinary healthcare professionals at the outpatient clinic will have access to participants’ personal data to enable them to provide patient care. The data extracted for the purposes of this study will be anonymised. All personal data extracted will be stored in the trust computers, which can only be accessed by research investigators and are password protected with restricted access to unauthorised individuals. The computers are encrypted and kept in a secure building with swipe card access. Access to the computers will be via a secure login. All handling, processing, and storage of personal identifiable data and study data will be in accordance with the Data Protection Act 1998 and the NHS Code of Confidentiality.

We will store research data generated by the study for 5 years at UCL. The chief investigator will have long-term access to research data after the study has ended.

### Statistical analysis

All analysis will be conducted according to the intention-to-treat principle with the use of SPSS version 22 (IBM, Armonk, NY, USA). Patients are evaluated for analysis if they received a study treatment. If the clinical course cannot be fully evaluated, the last point of visit is considered as the last data analysed. Baseline characteristics of the two groups will be recorded. The continuous variables will be compared using Student’s t-test. The categorical variables will be compared using Pearson’s chi-square or Fisher’s exact test depending on the number of events.

The proportion of wounds healed with each treatment will be compared using a chi-square test or Fisher’s exact tests, depending on the number of events. Mean time to wound healing will be determined on the basis of the number of days until complete re-epithelialisation, using Kaplan-Meier analysis of cumulative wound healing, followed by a log rank test.

Secondary outcomes will be compared between groups using a chi-square test for categorical variables. Non-normally distributed continuous variables will be compared using a Mann-Whitney U test. A *p* value of less than 0.05 will be considered significant, and all tests will be two-sided.

## Discussion

Wound care presents a significant financial and resource burden to the healthcare system, with about £5.0 billion spent annually in caring for patients with wounds in the United Kingdom alone. This signifies a need to further optimise the current wound coverage strategies [11, 12]. EG for wound healing is not a new concept, and several case reports have reported a good wound healing outcome; however, it is not known if the healing rate is comparable to SSG, a mainstay of treatment for wounds that cannot be closed primarily [2, 5, 13].

EPIGRAAFT is designed as a randomised, controlled, parallel-group, multicentre study to investigate the efficacy of EG against SSG. Our hypothesis is that EG has the same wound healing outcome with SSG but with lower donor site morbidity. This trial design is pragmatic with scientific evaluation on the wound-healing mechanism by EG and SSG to promote further understanding and compare the mechanism of healing at the cellular level. It is postulated that EG stimulates wound healing by acting like a bioengineered skin by expressing growth factors, thereby encouraging the wound bed to regenerate, and initiates keratinocyte migration from the edges of the wound [2]. In vitro studies showed that the migrating keratinocytes from the grafts synthesise several growth factors, namely the vascular endothelial growth factor, hepatocyte growth factor, granulocyte colony-stimulating factor, platelet-derived growth factor, and transforming growth factor α [14, 15]. The migrating keratinocytes also deposit a variety of extracellular matrix components, such as laminin, fibronectin, and type IV collagen [16]. The wound exudate analysis in this trial will demonstrate the expression pattern of the growth factors expressed by the grafts in vivo. The effect of the growth factors on the wound bed and the edges of the wound will be further confirmed by the punch biopsies. The punch biopsy taken from the centre of the wound will be used to identify the expression pattern of the keratinocyte proliferative markers before and after treatment, which could suggest the activation of the wound bed with treatment. The skin biopsy from the wound edge will be used to study the migratory activity of the keratinocytes by determining the expression pattern of the Connexin proteins. The Connexin proteins are gap junctional proteins which are channel-forming proteins enabling adjacent cells to communicate and play a vital role in coordinating cell proliferation and migration [17]. It is known that the downregulation of Connexin protein at the edges of the wound correlates with increased keratinocytes migratory activity, resulting in accelerated wound healing [17]. As the EG is postulated to stimulate the keratinocytes at the wound edges to proliferate and migrate onto the wound bed, downregulation of the Connexin protein is expected at the wound edges. This, in turn, indicates that the keratinocytes have increased migratory properties. This will also be correlated with the proliferation markers of keratinocytes.

This study is expected to define the efficacy of EG and further understand the mechanism of wound healing by EG compared to SSG. These results can be used to inform the current best practise for wound care.

## Trial status

At the time of manuscript submission, the trial was actively enrolling participants.

### Dissemination policy

The results of the study will be reported and disseminated in peer-reviewed scientific journals, conference presentations, and website/online publications, as well as in internal reports. Reporting will be based on CONSORT guideline for reporting randomised trials. All publications will be forwarded to participants.

## Additional files

Additional file 1: SPIRIT 2013 Checklist: Recommended items to address in a clinical trial protocol and related documents*. (DOC 120 kb) Additional file 2: Patient Consent Form. (DOCX 49 kb)

## Abbreviations

CONSORT: Consolidated Standards of Reporting Trials
CRF: clinical research folder
DAE: device-related adverse event
EG: epidermal grafting
H&E: haematoxylin and eosin
NHS: National Health Service
NHS R&D: National Health Service Research and Development
NPWT: negative pressure wound therapy
RFH: Royal Free Hospital
SPIRIT: Standard Protocol Items: Recommendations for Interventional Trials
SAE: severe adverse event
SSG: split-thickness skin grafting
UCL: University College London
WAE: wound-related adverse event
