# Supplementary material for: Epidermal grafting versus split-thickness skin grafting for wound healing (EPIGRAAFT): study protocol for a randomised controlled trial
Source: Trials. 2016 May 17;17:245. doi: 10.1186/s13063-016-1352-y (PMC4869340; doi:10.1186/s13063-016-1352-y)
Supplement: Additional file 2: — Patient Consent Form. (DOCX 49 kb) [file 13063_2016_1352_MOESM2_ESM.docx]

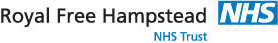


**CONSENT FORM**

Study Title: **A Randomised Controlled Trial to Evaluate the Efficacy of Epidermal Grafting in Wound Healing (EPIGRAAFT)**

Form version: May 11^th^ 2015, Version 1.1 Project ID number:

Chief Investigator : **Professor Toby Richards/ Mr Afshin Mosahebi**

**To be completed by the volunteer Please initial each box**

| 1) I confirm that I have read and understood the information sheet dated May 11^th^ 2015, version 1.1 for the above study, and have had the opportunity to ask questions. |  | |
| --- | --- | --- |
|  |  | |
| 2) I confirm that I have had sufficient time to consider whether or not I want to be included in the study. |  | |
|  |  | |
| 3) I understand that my participation is voluntary and that I am free to withdraw at any time, without giving any reason, without my medical care or legal rights being affected. |  | |
|  |  | |
| 4) I understand that relevant sections of my medical notes and data collected during the study may be looked at by investigators of the trial, from regulatory authorities or from the NHS Trust, where it is relevant to my taking part in this research. I give permission for these individuals to have access to my records (which may include them being sent a copy of this consent form). |  | |
|  |  | |
| 5) I agree to biopsies to be taken at the wound bed and the wound edge at the start of the treatment and at day 7. |  | |
|  |  | |
| \| 6) I understand that I am gifting my tissue to the investigators and in doing so I give up all future claims to its use that may include further research. \|  \| \| --- \| --- \| \| 7) I agree that my GP can be informed of my involvement in this study. \|  \| \| 8) I agree that anonymous clinical photographs may be taken of my wound during this study at each follow-up. \|  \| \| 9) I agree to take part in the above study. \|  \| | |  |

_______________________ _________________________ _______________

Name of patient Signature Date

________________________ _________________________ _______________

Name of person taking Signature Date

consent (if different from researcher)

________________________ _________________________ _______________

Researcher Signature Date

*When completed: 1 form for patient, 1 to be kept as part of the researcher site file, 1 to be kept with hospital notes.*

**Contact details for further information**

Professor Toby Richards, Professor of Surgery & Consultant Vascular and Endovascular Surgeon, Email: [toby.richards@ucl.ac.uk](mailto:toby.richards@ucl.ac.uk) Tel: 0207 6796454

Mr Afshin Mosahebi, Consultant Plastic Surgeon and Honorary Senior Lecturer, Email: [a.mosahebi@ucl.ac.uk](mailto:a.mosahebi@ucl.ac.uk) Tel: 020 77940500 ext 35556
